# The Relationship Between Perceived Social Support and Spiritual Well‐Being in Patients With Multiple Sclerosis

**DOI:** 10.1002/nop2.2204

**Published:** 2025-05-13

**Authors:** Fatemeh Nazari, Vahid Shaygannejad, Leila Mardanian Dehkordi

**Affiliations:** ^1^ Nursing and Midwifery Care Research Center Isfahan University of Medical Sciences Isfahan Iran; ^2^ Isfahan Neurosciences Research Centre, Neurology Department, School of Medicine Isfahan University of Medical Sciences Isfahan Iran

**Keywords:** multiple sclerosis, perceived social support, spirituality

## Abstract

**Aim:**

This study aimed to determine the relationship between spiritual well‐being and perceived social support (PSS) in patients with multiple sclerosis (MS).

**Design:**

This was a cross‐sectional study.

**Patient Contribution:**

This study was conducted on 120 patients with MS in Isfahan in 2021. The participants were over 18 years of age and referred to the MS Clinic.

**Methods:**

The subjects were selected using convenience sampling. Data were collected using Cheraghi and Davari Dolatabadi's Perceived Social Support and Paloutzian and Ellison's Spiritual Well‐Being (SWB) questionnaires and analysed using descriptive (number and percentage, mean and standard deviation) and inferential (Pearson's test, linear regression) statistics in SPSS software.

**Results:**

Pearson correlation test indicated that there was a direct relationship between spiritual well‐being and the total score of perceived social support (*r* = 0.415, *p* < 0.001) and the emotional (*r* = 0.328, *p* = 0.004) and informational (*r* = 0.311, *p* = 0.006) dimensions; this relationship was not significant in the instrumental dimension (*r* = 0.197, *p* = 0.081). Moreover, linear logistic regression analysis showed that in MS patients, the chance of spiritual well‐being among MS patients will be increased by 59.2% per unit increase in emotional support. Also, the chance of spiritual well‐being increases by 34.1% for each score increase in the age of MS patients.

**Conclusions:**

It is necessary that age and perceived emotional support, as a strong predictor of spiritual health status, are considered in designing health promotion interventions for patients with MS.

## 1 INTRODUCTION

Multiple sclerosis (MS) is a chronic autoimmune central nervous system (CNS) disease that is associated with the demyelination of axons in the brain and the spinal cord, leading to progressive neurological damage and a set of disabling symptoms (Strayer Andrea 2020; Akaishi et al. 2020; Huang et al. 2017). Multiple sclerosis can occur at any age, but the highest prevalence is in people between 20 and 50 years old (Hinkle and Cheever 2018), and around 2.8 million people worldwide are affected by MS (Walton et al. 2020). The incidence of this disease in Isfahan, Iran, was reported to be 71.6 per 100,000 people. However, the reason is not known, but Isfahan can be recognised as an area with a medium to high risk of MS (Bagherieh et al. 2023). MS often leads to an early or overall reduction in the physical, social, and cognitive functions of the individual, and affects the quality of life (QOL) of the patient and his/her family (Strober 2018). The key factor affecting QOL is health status. Health has biomedical, social, psychological, anthropological and spiritual dimensions (van Druten et al. 2022). Spiritual health is one of the basic aspects of health, which is considered an important approach to promoting health by creating meaning and purpose in life (Jaberi et al. 2019). There was a meaningful relationship between spirituality and a person's overall health, and hope as a factor in relation with spiritual health that can have positive and useful effects on the improvement of MS patients (Niyazmand et al. 2018). However, evidence showed that the spiritual health of MS patients is at a moderate level (Shahabian et al. 2020).

Social support may play an important role in maintaining physical (Mardanian‐Dehkordi and Kahangi 2018) and psychosocial well‐being (Razeghi et al. 2024). Social support has been defined as an individual's understanding or experience of love, companionship, care, respect, attention, and assistance received by others, as mutual assistance and commitments on the part of a social network (Alorani and Alradaydeh 2018). Social support, as an emotional coping method, can protect people by preventing stressful situations such as chronic diseases and help them to assess stressful events in such a way that they are less threatening (Calhoun et al. 2022).

Although the issue of social support has been less discussed, it is one of the areas related to nursing that embraces a wide range of life aspects, plays a vital role in promoting adaptation to chronic diseases, and improves healing outcomes (Nikbakht Nasrabadi et al. 2021a; Nikbakht Nasrabadi et al. 2021b). Nurses can act as a source of support for family caregivers in a variety of ways, such as facilitating economic support for the families of patients, mobilising the social support networks of the client and establishing a relationship between the client and the relevant social resources (Reinhard and Brassard 2020). Nurses can provide informational support by providing clients with information about self‐care or educating members of the client's social network (Nikbakht Nasrabadi et al. 2021a; Nikbakht Nasrabadi et al. 2021b). Studies show that the identification of perceived social support by patients can be effective in promoting their adaptive behaviours and providing appropriate community support (Harooni et al. 2014). A review of studies showed that, in most studies, there was a significant relationship between perceived social support and mental and social well‐being, meaning that perceived social support has a positive impact on mental, physical and social health (Harooni et al. 2014). The existence of social support in individuals, in addition to the reduction of blood pressure, neurotic headaches, gastrointestinal disorders, etc., also enhances self‐esteem and dignity in individuals (Arabshahi et al. 2020).

In general, the above points show that social support and positive social relationships have positive effects on the physical, socio‐psychological and economic well‐being of patients. They also reduce the severity of disease symptoms, improve the patient's QOL, feeling towards life and ability to cope with the disease and create a better public evaluation of life (Smith and Mackie 2014). On the other hand, it was pointed out that the level of spiritual health is not high in patients with MS (Shahabian et al. 2020), and comprehensive attention to the health status of patients with MS is considered to be one of the requirements of the health system of each country. Thus, in order to identify the factors related to spiritual well‐being, and considering the various effects of social support on various aspects of life, researchers tried to examine the relationship between these two concepts.

A relationship that, if present, can be exploited to strengthen the spiritual well‐being of patients with MS. Most nursing models emphasise the holistic approach to care, and nurses are always urged to be committed to the concept of holistic care and, in addition to the physical, mental, emotional and social needs of patients, to recognise their spiritual needs and support them. Given the fact that MS affects the way a person lives, it causes many problems in all physical, mental, social, economic and familial aspects, and causes patients to be dependent on others and less capable of supporting others. Moreover, they cannot participate in common social activities. Therefore, according to the mentioned materials, it seems necessary to focus on the concept of spirituality and spiritual health as an aspect that has been paid less attention to and investigate its relationship with perceived social support in chronic diseases.

### 1.1 Research Question

Is there a significant relationship between spiritual well‐being and perceived social support for patients with MS?

## 2 MATERIALS AND METHODS

### 2.1 Study Design and Population

This cross‐sectional study was performed on 120 women suffering from MS from 6th July 2021 to 18th November 2021. The women were over 18 years of age and referred to the specialised MS Clinic of Kashani Hospital, Isfahan. The inclusion criteria included suffering from MS (recurrent, primary progressive and secondary progressive), definitive diagnosis of MS by a neurologist based on Revised McDonald's Criteria of 2010 (Polman et al. 2011), the passage of at least 1 year since diagnosis, lack of any attacks and relapse in the past 3 months, Persian speaker, literacy, willingness to participate in the study, the absence of physical defects such as Wernicke's aphasia or cognitive defects that are the cause of incorrect responses to the questions and an Expanded Disability Status Scale (EDSS) score of < 5.5. In addition, the exclusion criteria included unwillingness to continue cooperation and complete the questionnaire. The sample size was calculated using the estimate of the correlation coefficient between the spiritual health score and perceived social support score of at least 0.25 (Harooni et al. 2014), a confidence interval of 0.95 and test power of 0.84.

### 2.2 Sampling Procedure

The participants were selected using the convenience sampling method. After obtaining permission from the research deputy of Isfahan University of Medical Sciences, Iran, and the officials of Kashani Hospital, the researcher began sampling from among the patients who were referred to the MS Clinic of this hospital. Then, the objectives of the study, the study method, and the subjects' rights were explained to the participants and they were asked to sign an informed consent form. Then, the questionnaire was distributed among them, and the necessary explanations were provided. All data was collected through interviews to prevent bias by an interviewer.

### 2.3 Study Instrument

The data collection tool used in this study consisted of a demographic characteristics form (including age, gender, marital status, educational level and clinical trend of the disease), and the EDSS and Spiritual Well‐Being Scale (SWBS) developed by Paloutzian and Park (2014). The SWB includes 20 questions scored based on a 6‐point Likert scale ranging from totally agree to totally disagree. This scale is divided into two subscales of religious and existential well‐being, each of which includes 10 items with a total score of 10–60. Odd items show religious well‐being and even items indicate existential well‐being. The total score on the scale was the sum of these two subscales, which ranges between 20 and 120. According to the scores obtained, spiritual well‐being is divided into high (100–120), moderate (41–99) and low (20–40) levels. The reliability and validity of the SWBS were standardised by Biglari Abhari et al. (2018) in a research paper entitled ‘Validation of the Persian version of spiritual well‐being Questionnaires’. This tool has been used in several studies to measure spiritual well‐being in Iran (*α* = 0.70; Abbasi et al. 2014; Shahabian et al. 2020). In the current study, the reliability of this tool and its two dimensions (religious and existential well‐being) was obtained using Cronbach's alpha coefficient which was, respectively, 0.79, 0.76 and 0.78. Perceived social support was measured using the Perceived Social Support Inventory developed by Cheraghi and DavariDolatabadi (2016) in the three dimensions of emotional support, informational support and instrumental support. Emotional support includes the emotions of empathy, love, affection, trust, acceptance of the patient and respect for the patient. Informational support includes information or advice that can help the person adapt to problems and solve them. The instrumental support includes tangible assistance including the provision of services, assistance in activities, financial support and other assistance provided to the client.

The questions of the perceived social support are scored based on a scale of repetition of action ranging from never (1 point) to always (4 points). The total score of the scale is the sum of these points and is in the range of 30–120. A higher score indicates a greater perception of support by the patient for each question. According to the obtained scores, perceived social support was divided into high (higher than 90), medium (60–90) and low (less than 60) levels. The validity and reliability of this tool were determined through content validity and Cronbach's alpha (*α* = 87%), respectively. To evaluate the reliability of the Perceived social support in this study, its internal consistency was determined using Cronbach's alpha, which was, respectively, 0.89, 0.85, 0.89, and 0.82 for total social support and the emotional, informational and instrumental support structures. To determine the EDSS score, the functions of the extrapyramidal, cerebellum, brainstem, sensory, urinary and excrement, visual and brain systems must be investigated. The total score of this scale ranges between 0 and 10. A score of 0, 1–4.5, 5–5.5, 6, 6.5, 7–9.5 and 10, respectively, signifies a normal nervous condition, a person who is totally competent and does not require help, inability to perform daily activities, the need for one‐sided support for the patient, a need for mutual support, significant damage in the organs and the need for a wheelchairs and 10 points represent death due to MS.

### 2.4 Data Analysis

After completing the questionnaires, the collected data were analysed using SPSS software (version 18.0, SPSS Inc., Chicago, IL, USA). The significance level in all tests was considered to be < 0.05. Descriptive (number and percentage, mean and standard deviation) and inferential (Pearson's test, linear regression) statistics were used for data analysis to reach the research goals and answer the research questions. As correlation coefficient between spiritual well‐being and the total score of social support and its dimensions was determined by Pearson's test. Variables of social support affecting spiritual well‐being were analysed by multivariate logistic regression.

### 2.5 Ethical Considerations

This research was approved with the ethical code IR.MUI.REC.1400.2.143. Then, written informed consent was obtained from all patients and assured that their names and information would remain confidential and that they could withdraw from the research whenever they wished.

## 3 RESULTS

Of the 135 completed questionnaires, 120 questionnaires could be analysed (response rate = 90%). The mean disease duration and age of participants were 8.1 (5.9) years and 33.67 (8.7) years, respectively, and 70.8% were women and 74.2% were married. In addition, 44.2% had a university degree, 48.4% were housewives and 61.7% had a clinical pattern of recurrence and recovery (Table 1).

**TABLE 1:** Distribution of the demographic characteristics of subjects.

Variable	Number (percentage)
Type of clinical course	
RRMS	74 (61.7)
PPMS	18 (15.0)
SPMS	28 (23.3)
Total	120 (100)
Employment status	
Unemployed	13 (10.9)
Housewife	58 (48.4)
Student	14 (11.6)
Worker	5 (4.1)
Retired	4 (3.4)
Employee	18 (15.0)
Business	8 (6.6)
Total	120 (100)
Level of education	
Illiterate	3 (2.5)
Elementary	8 (6.7)
Guidance school	19 (15.8)
High school	37 (30.8)
University degree	53 (44.2)
Total	120 (100)
Marital status	
Single	31 (25.8)
Married	89 (74.2)
Total	120 (100)
Sex	
Female	85 (70.8)
Male	35 (29.2)
Total	120 (100)
Age(years)	
Mean = 33.67 SD = 8.7	

Abbreviations: PPMS, primary progressive multiple sclerosis; RRMS, relapsing–remitting multiple sclerosis; SPMS, secondary progressive multiple sclerosis.

The findings of the present study showed that the average total spiritual well‐being score of participants was 85.02 (20.77). The average scores for religious well‐being and existential well‐being were 47.12 (9.88) and 38.45 (11.74), respectively. The spiritual well‐being level of 26.2% of the subjects was at a high level, 69.7% at an average level and 4.1% at a low level. There was no statistically significant relationship between age, gender, marital status, education level, duration of MS disease and clinical course pattern with spiritual health (*p* > 0.05; Table 2).

**TABLE 2:** The relationship between spiritual health and some individual and clinical factors.

Variable	Total spiritual health score		
	*r*	*t* or *F*	*p*
Age	0.130	—	0.255
Duration of MS disease	0.068	—	0.580
Sex	—	0.053	0.958
Marital status	—	0.584	0.561
Level of education	—	0.709*	0.589
Clinical course pattern	—	1.449*	0.242

*Note:* Level of significance at *p* ≤ 0.05.

Abbreviations: *r*, Pearson correlation coefficient; *t*, independent sample *t*‐test.

* *F* = one‐way ANOVA.

The mean total score of perceived social support of the participants was 62.19 (14.55); 47.1%, 49.6% and 4.1% were, respectively, at low, average and high levels. The average scores for emotional, instrumental and informational support were 30.09 (7.84), 17.6(5.11) and 14.87 (4.23), respectively. The findings of the present study indicate that there is a direct relationship between spiritual well‐being and the total score of perceived social support (*r* = 0.236, *p* = 0.009) and the emotional support subscale (*r* = 0.314, *p* = 0.001) but there is no relationship between spiritual well‐being and the subscales of informational (*r* = 0.161, *p* = 0.094) and instrumental support(*r* = 0.132, *p* = 0.173). Also, there was no significant relationship between age and the total score of perceived social support and the instrumental support subscale (*p* > 0.05) but there was an indirect relationship between age and the subscales of informational (*r* = 0.955, *p* = 0.006) and emotional support(*r* = 0.933, *p* = 0.009) in patients with MS. There was no statistically significant relationship between the duration of MS disease and total perceived social support and its emotional and instrumental dimensions (*p* > 0.05). However, there was a statistically significant relationship between the duration of MS disease and the informational dimension of perceived social support (*t* = −0.229, *p* = 0.041).

Furthermore, there was a direct relationship between sex and the information subscale of perceived social support (*t* = 3.310, *p* = 0.002) but there was no relationship between sex and the total score of perceived social support and its emotional and instrumental subscales (*p* > 0.05). There was a direct significant relationship between marital status and the total score of perceived social support (*t* = 2.205, *p* = 0.030) and instrumental support subscale (*r* = 2.794, *p* = 0.006) but there was no relationship between marital status and the subscales of informational and emotional support in patients with MS (*p* > 0.05). Also, there was no statistically significant relationship between the level of education and clinical course pattern with the total score of perceived social support and its dimensions (*p* > 0.05; Table 3). Moreover, linear logistic regression analysis showed that in MS patients, the chance of spiritual well‐being among MS patients will be increased by 59.2% per unit increase in emotional support. Also, the chance of spiritual well‐being increased by 34.1% for each score increase in the age of MS patients (Table 4).

**TABLE 3:** The relationship between social support and its dimensions with spiritual well‐being and some individual and clinical factors.

Dimensions of social support	Total social support			Emotional support			Informational support			Instrumental support		
Variable	*r*	*t* or *F*	*p*	*r*	*t* or *F*	*p*	*r*	*t* or *F*	*p*	*r*	*t* or *F*	*p*
Spiritual well‐being	0.236	—	0.009	0.314	—	0.001	0.161	—	0.094	0.132	—	0.173
Age	−0.030	—	0.784	−0.933	—	0.009	−0.955		0.006	−0.562		0.059
Duration of MS disease	−0.158		0.193	−0.110		0.339	−0.229		0.041	−0.009		0.938
Sex	—	1.596	0.114	—	1.409	0.162	—	3.310	0.002	—	0.438	0.663
Marital status	—	2.205	0.030	—	1.682	0.096	—	2.056	0.05	—	2.794	0.006
Level of Education	—	0.636*	0.639	—	0.890*	0.473	—	1.761*	0.143	—	0.628*	0.646
Clinical course pattern	—	1.451*	0.241	—	2.610*	0.08	—	1.458*	0.239	—	1.002*	0.372

*Note:* Level of significance at *p* ≤ 0.05.

Abbreviations: *r*, Pearson correlation coefficient; *t*, independent sample *t*‐test.

* *F* = one‐way ANOVA.

**TABLE 4:** Linear regression coefficients of age, disease duration and the dimensions of social support variables on spiritual well‐being.

Spiritual well‐being	Unstandardised coefficients		Standardised coefficients	*t*	*p*
	*β*	SE	*β*		
1 (Constant)	20.345	12.906		1.576	0.120
Emotional support	1.519	0.365	0.592	4.160	<0.001
Informational support	−0.746	0.740	−0.155	−1.007	0.318
Instrumental support	0.168	0.465	0.048	0.362	0.718
Age	0.876	0.321	0.341	2.724	0.008
Duration of MS disease	0.045	0.418	0.014	0.107	0.915

## 4 DISCUSSION

The present study aimed to investigate the status of spiritual well‐being and its relationship with perceived social support (PSS) in patients of MS. According to the findings, regarding spiritual well‐being, most patients with MS had average spiritual well‐being, which was consistent with the study by Shahabian et al. (2020). In fact, spirituality is an important source of power and support throughout life, helping to escape critical and stressful situations, and high spiritual well‐being indicates that other dimensions of human existence are in balance (Bożek et al. 2020). Our data indicated no significant relationships between age, gender, marital status, educational level, duration of MS disease and total spiritual well‐being score. Similarly, a study reported no significant relationship between age, gender, marital status, educational state and spiritual well‐being (Ahmadpoori and Motaghi 2020).

Regarding, an average level of perceived social support was observed in 49.6% of subjects, with a mean score of 62.19 (14.55), and this finding was consistent with the studies of Maghbooli et al. (2022). However, the study by Papa et al. (2021) reported a high perceived social support of median: 65 (IQR: 58–74; Papa et al. 2021). This difference could be due to the cultural differences between Iran and Greece. Although the findings of a study showed that low perceived social support is a predictor of MS (Dębska et al. 2020). Patients with MS have less social communication due to multiple physical and spiritual symptoms and their impact on all dimensions of QOL, and with decreasing social networks, their social support sources decrease. Therefore, their perceived social support is diminished. On the other hand, with the prolongation of the disease process, the communication network members gradually develop chronic fatigue and the level of social support provided decreases.

Our data indicated an indirect significant relationship between age and subscales of informational and emotional support and a significant direct relationship between gender and the information subscale of perceived social support. It means that age affects people's roles, needs, supportive relationships and social connections. Also, there was a direct significant relationship between marital status and the total score of perceived social support and the instrumental support subscale, which was consistent with the studies by Papa et al. (2021) and Maghbooli et al. (2022). The study findings showed that there were no statistically significant associations between gender and perceived social support. Also, Baharian et al. (2023) found no statistically significant associations between gender and perceived social support, too.

The study findings showed that there was a statistically significant relationship between the duration of MS disease and the information dimension of perceived social support, but there was no statistically significant relationship between the level of education and clinical course pattern with the total score of perceived social support and its dimensions. This finding was consistent with the study of Maghbooli et al. (2022). However, the findings of the study showed that perceived social support was associated with marital status, difficulties with social and family environment, modification of daily activities, help in daily activities, frequent urination, movement assistance, forgetfulness, belief in God and relations with health professionals (Papa et al. 2021).

Moreover, the findings of this study showed a significant positive relationship between the spiritual well‐being score and the total score of perceived social support and the emotional dimension of social support. However, the perceived emotional support and age are strong predictors of spiritual well‐being status in patients with MS, which was in agreement with the results of the study by Razeghi et al. (2024). The results of this research indicated that social support, through playing a mediating role between life stressors and the occurrence of physical and mental problems and the empowerment of individuals' cognition, reduces the experienced tension (Razeghi et al. 2024). Moreover, favourable social support leads the individual towards physical health and psychological and spiritual well‐being (Papa et al. 2021). The findings of Razeghi et al. (2024) showed that social support provided has a positive relationship with perceived mental health in patients with chronic disease (Razeghi et al. 2024), and social support provided by others has a significant direct relationship with general health dimensions based on the SF‐36 scale.

### 4.1 Limitations

Among the limitations of this study were data collection in one hospital and the small size of the statistical population and the study was carried out in one city. Therefore, it is recommended that future studies be carried out with a larger sample size in different cities with different cultures and in different age groups. The lack of cooperation from the subjects was another constraint of this study; despite the necessary explanations and obtaining the consent of participants for cooperation, 15 of the participants refused to cooperate. Furthermore, this data is derived from a cross‐sectional study, so the analysis cannot interpret causal relationships and longitudinal studies can help identify the cause and outcome of the relationship. Carrying out qualitative research in this respect is recommended in order to better understand how social support affects spiritual well‐being and its impact on improved QOL.

## 5 CONCLUSION

The level of perceived social support and its dimensions, especially the emotional dimension, had a positive relationship with the level of spiritual well‐being of the patients. Therefore, it is necessary that age and perceived emotional support, as a strong predictor of spiritual health status, are considered in designing health promotion interventions by the treatment team, especially the nurses for patients with MS.

## AUTHOR CONTRIBUTIONS

**Fatemeh Nazari:** concepts; design; definition of intellectual content; literature search; clinical studies; experimental studies; data acquisition; data analysis; statistical analysis; manuscript preparation; manuscript editing; manuscript review; guarantor. **Vahid Shaygannejad:** concepts; design; definition of intellectual content; clinical studies; data acquisition; manuscript preparation; manuscript editing; manuscript review; guarantor. **Leila Mardanian Dehkordi:** concepts; design; literature search; experimental studies; statistical analysis; manuscript editing; manuscript review; guarantor.

## CONFLICTS OF INTEREST

The authors declare no conflicts of interest.

## Data Availability

The data that support the findings of this study are available from the corresponding author upon reasonable request.
